# Recovery-Targeted Supplemental Oxygen Enhances Performance and Attenuates Perceived Fatigue During Subsequent High-Intensity Swimming

**DOI:** 10.3390/sports14030085

**Published:** 2026-02-24

**Authors:** Joshua A. Kidwell, Trent Yamamoto, Aidan Flanagan, Vishruth Shatagopam, Kyle J. Hetherton, Keegan Slomba, August Blatney, Jillian Smith, Eric V. Neufeld, Brett A. Dolezal

**Affiliations:** 1 Airway and UC Fit Digital Health-Exercise Physiology Laboratory, David Geffen School of Medicine, University of California Los Angeles, Los Angeles, CA 90095, USA; 2 School of Medicine, Creighton University, Omaha, NE 68124, USA; 3 Chobanian and Avedisian School of Medicine, Boston University, Boston, MA 02118, USA; 4 Northwell Orthopedics, New Hyde Park, NY 11042, USA; 5 Long Island Jewish Medical Center, North Shore University Hospital, New Hyde Park, NY 11030, USA

**Keywords:** supplemental oxygen, hyperoxia, recovery, swimming performance, aquatic athletes, perceived exertion, repeated exercise

## Abstract

High-intensity aquatic sports require athletes to repeatedly produce near-maximal efforts under conditions of constrained ventilation and limited recovery between bouts, placing substantial importance on recovery efficiency. While supplemental oxygen has been proposed as a recovery-targeted strategy to support repeated high-intensity performance, its acute effects in aquatic athletes remain poorly characterized. The purpose of this study was to examine whether brief inhalation of supplemental oxygen during recovery following a maximal swim effort influences subsequent swimming performance and perceived exertion in trained aquatic athletes. Eighteen collegiate-aged male aquatic athletes completed a randomized, placebo-controlled, within-subject crossover protocol. Each condition consisted of a maximal 100-yard (91.44 m) swim followed by a standardized recovery period that included a five second inhalation of either 98% supplemental oxygen or ambient air delivered via an identical portable device, prior to a maximal 50-yard (45.72 m) freestyle sprint. Sprint performance was significantly faster following oxygen-assisted recovery compared with placebo, and perceived exertion was significantly reduced at the post-exercise time point, with no differences observed prior to exercise or mid-protocol. These findings suggest that brief, recovery-targeted hyperoxia may enhance repeated high-intensity swimming performance while attenuating post-exercise perceived exertion in trained aquatic athletes.

## 1. Introduction

Success in high-intensity aquatic sports, such as water polo and competitive swimming, requires athletes to repeatedly generate near maximal power and performance under conditions of constrained ventilation, effective oxygen uptake, and limited recovery between each effort [1]. Unlike land-based exercises, swimming and water exercises impose unique cardiopulmonary and ventilatory demands and restrictions due to altered pulmonary mechanics under hydrostatic pressure, breath-holding patterns, and their resulting delays in carbon dioxide clearance [2,3]. Each alone can exacerbate ventilatory strain and accelerate fatigue during training and competition. Consequently, outcomes in these activities are determined not solely by muscular strength/power and metabolic capabilities, but also by the effectiveness of recovery mechanisms and the athlete’s ability to withstand respiratory and perceptual stress during successive intervals of maximum exertion.

In swimming training and competition, performance is strongly influenced by the rate at which intramuscular energy stores, particularly phosphocreatine (PCr), are restored during brief recovery intervals [4]. PCr resynthesis is an oxygen-dependent process that most rapidly occurs within the first thirty to sixty seconds following intense exercise [5,6,7,8]. Previous research has shown incomplete PCr restoration directly impaired subsequent exercise and sprinting performances under conditions of limited recovery, and similar constraints are present during repeated maximum-effort swim repeats [5,6,7,8]. Accordingly, interventions capable of enhancing oxygen availability during the early recovery period may improve the ability to sustain performance across repeated high-intensity efforts, particularly in aquatic sports.

In this context, the addition of supplemental oxygen during rest periods has been explored as a potential ergogenic strategy to enhance recovery based on its ability to acutely increase the fraction of inspired oxygen and transiently elevate arterial oxygen tension during recovery [9,10]. Although at sea level most healthy individuals will have near maximum hemoglobin saturations, hyperoxic breathing can readily increase the amount of dissolved oxygen in plasma and thereby enhance tissue oxygen availability independent of minimal changes in hemoglobin binding capacity [6,9]. Recent experimental and meta-analytic evidence further supports that short-duration hyperoxic exposure can improve repeated-sprint performance and recovery kinetics across a range of land-based exercise modalities [9,10,11,12]. Consistent with this, prior works in land-based exercise models have demonstrated that breathing oxygen-enriched air during recovery can accelerate post-exercise metabolic recovery, enhance PCr resynthesis, and attenuate ventilatory strain during subsequent high-intensity efforts [6,10]. These findings collectively suggest that oxygen supplementation may be most relevant when applied during brief recovery periods, and may be particularly relevant in exercise modalities characterized by constrained ventilation and repeated bouts of intense efforts such as swimming.

Despite growing interest in portable supplemental oxygen devices among athletes and coaches, limited research has examined the acute effects of oxygen supplementation in aquatic sports or under conditions that closely replicate competitive swimming. Most prior investigations have focused on land-based exercise or clinical applications, resulting in uncertainty regarding whether brief hyperoxic exposure during recovery can meaningfully influence subsequent performance and/or perceptual responses in swimming modalities, where ventilation is constrained and recovery between efforts is often brief [1,2]. However, no prior studies have examined hyperoxic recovery specifically between repeated maximal swim sprints, despite established performance declines from incomplete metabolic recovery in swimming sprint protocols [7,8]. To address this, the current study examined the acute effects of brief supplemental oxygen inhalation during the recovery period following a maximal swim effort on a subsequent short distance maximal effort swimming performance and ratings of the perceived exertion in trained aquatic athletes. We hypothesized that hyperoxic recovery through oxygen supplementation during early recovery would improve subsequent performance and reduce perceived exertion during the effort compared with a placebo condition.

## 2. Materials and Methods

### 2.1. Study Design

This study employed a randomized, placebo-controlled, within-subject crossover design to examine the acute effects of brief supplemental oxygen inhalation during recovery from maximal effort swimming on subsequent maximal effort swimming performance and rating of perceived exertion (RPE) throughout the subsequent effort. Each participant completed two conditions, supplemental oxygen and placebo, in counterbalance order. All testing procedures were identical across conditions and were completed within a single testing session per condition.

### 2.2. Participants

**Figure 1:**
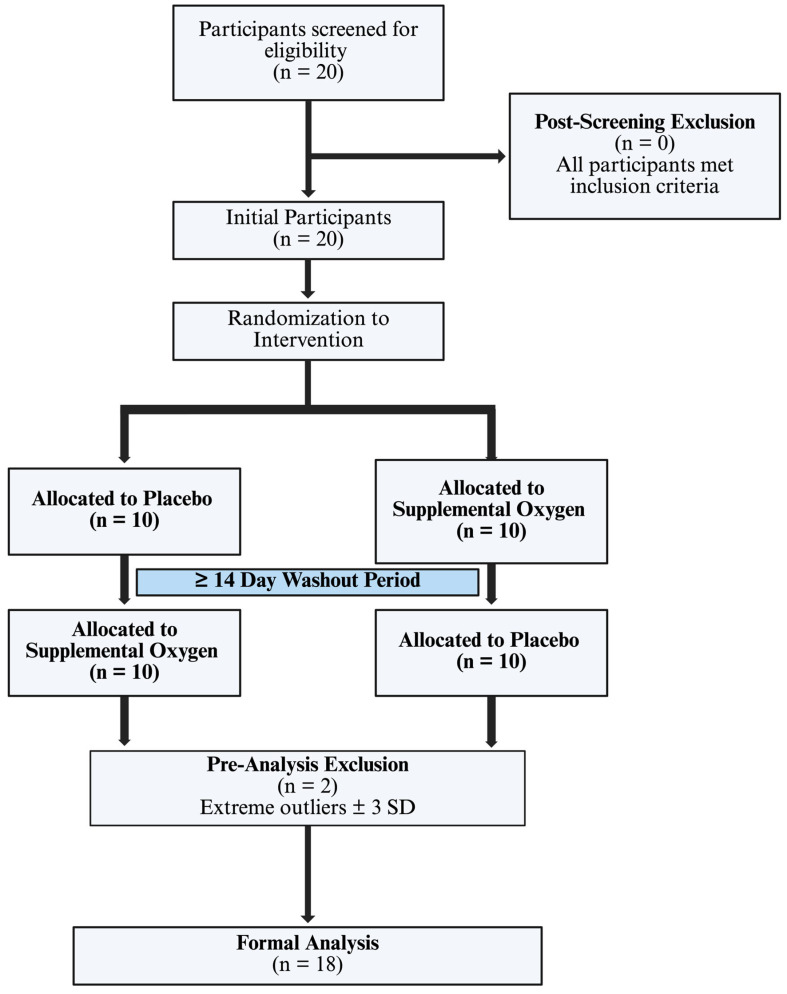
CONSORT diagram. Made with Biorender (2026).

Twenty trained male collegiate-aged aquatic athletes were enrolled (mean age = 20.5 ± 1.4 years, range: 18–23 years), all recruited from competitive swimming and water polo programs in the Los Angeles, CA area with a minimum of six years of structured aquatic training (≥1 session/week). Following pre-specified data screening for extreme outliers (see Supplementary Methods S1), the final analytic sample consisted of 18 participants. All participants met inclusion criteria of active structured aquatic training and ability to safely perform maximal swimming efforts. Exclusion criteria included known cardiopulmonary disease, contraindications to high intensity exercise, acute illness or musculoskeletal injury at the time of testing, or the inability to tolerate the breathing apparatus used during intervention and placebo conditions. Demographics and anthropometrics were recorded at baseline and are available in Table 1. Written informed consent was obtained from all participants, ethical approval was obtained from the University of California, Los Angeles (UCLA) (IRB: 11-003190) and single IRB approval for off-site participants (sIRB: BRANY, NY, USA). Research was conducted in accordance with the Declaration of Helsinki. Participant enrollment, allocation, exclusions, and the final analytic sample are summarized in Figure 1.

### 2.3. Experimental Setting

All testing sessions were conducted in a standard 25-yard (22.86 m) indoor competition pool maintained as consistent environmental conditions throughout testing. Participants were instructed to refrain from exercise and alcohol consumption for at least 24 h prior to testing and to maintain their regular dietary and hydration practices before each session. When possible, testing sessions were scheduled at similar times of day (i.e., morning) to minimize potential diurnal variations.

### 2.4. Experimental Protocol

Participants completed two conditions separated by a minimum two-week washout period to minimize any acute physiological or perceptual carryover effects. At the beginning of each session, participants completed an identical standardized warm-up before each testing session, consisting of dryland dynamic stretches (30 s each: forward/backward arm circles, torso twists, leg swings) followed by in-pool swimming: 200 yards easy pace, 4 × 50 yards build on 1:30, 2 × 25 yards fast on 1:00, and 50 yards easy scull down and back. Subsequently participants performed a maximal effort 100-yard (91.44 m) swim to induce fatigue. Immediately following this effort, participants underwent a standardized recovery period during which the designated intervention was administered: five seconds of intervention followed by a twenty-second rest. After completing the recovery interval, participants performed a maximal 50-yard (45.72 m) freestyle swim, which functioned as the primary measure of performance outcome.

### 2.5. Intervention Conditions

#### 2.5.1. Supplemental Oxygen

**Figure 2:**
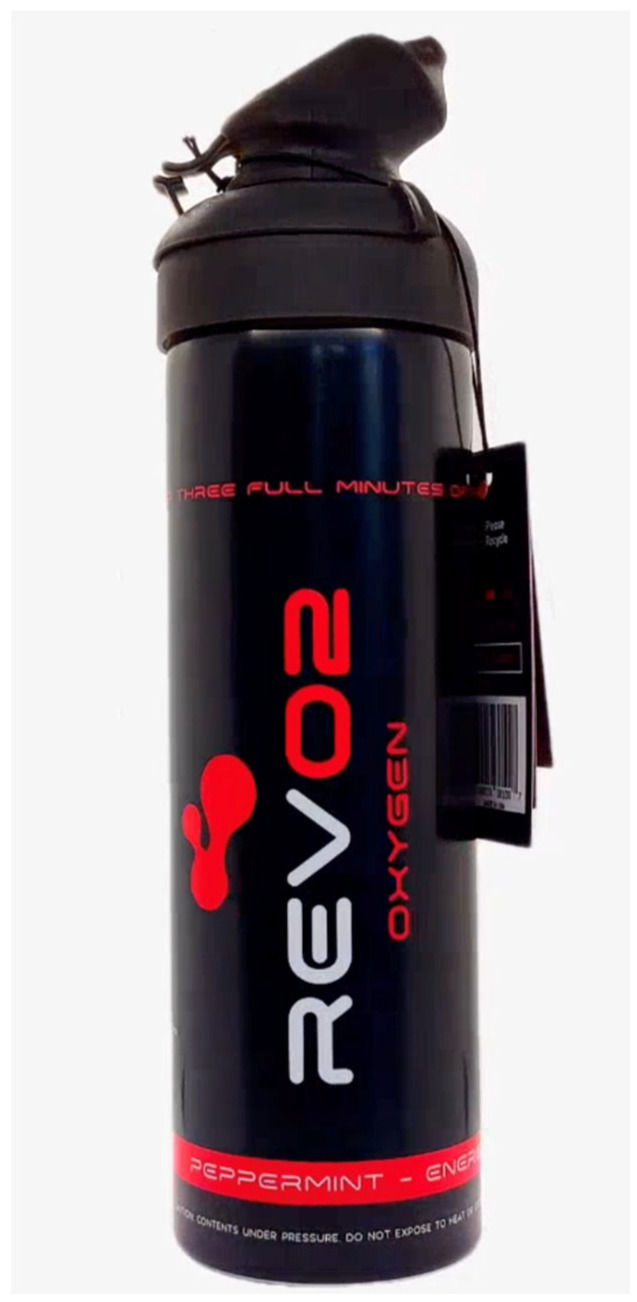
RevO2 Supplemental Oxygen Canister used as intervention.

During the recovery phase, participants received 98% compressed supplemental oxygen via a portable canister (RevO2 Supplemental Oxygen: RevO2, Irvine, CA, USA) administered through a standardized mouthpiece for exactly five seconds. Oxygen delivery commenced immediately upon completion of the swim from the water. The delivery device consisted of a cylindrical canister comparable in size to a standard water bottle and was equipped with a nozzle and push-trigger mechanism to allow controlled initiation and termination of airflow (Figure 2). The specific model utilized was Peppermint Oxygen- Energy and Endurance, infused with peppermint.

#### 2.5.2. Placebo Oxygen

During the placebo condition, participants used an identical delivery apparatus containing an empty canister that delivered ambient air without supplemental oxygen. To enhance blinding and reduce participants’ ability to distinguish conditions, the placebo canister was infused with peppermint extract, producing a similar taste and sensory experience to the supplemental oxygen condition without affecting oxygen intake [11]. Breathing instructions and intervention duration were identical across conditions.

Participants were not informed of the oxygen concentration associated with either condition. Outcome assessment procedures were standardized across conditions to minimize potential bias.

### 2.6. Outcome Measures

#### 2.6.1. Swimming Performance

The primary outcome measure was completion time for the 50-yard maximal effort swim performance after the intervention and recovery interval. Swim times were recorded using manual timing with a handheld stopwatch by a trained investigator following standardized timing procedures, a method that has demonstrated high reliability for measuring short-duration sprint performance [12]. Timing began at the initiation of movement and ended upon completion of the 50-yard distance. Times were recorded in seconds to the nearest hundredth and analyzed as continuous variables.

#### 2.6.2. Ratings of Perceived Exertion

Perceived exertion was assessed using the Borg REP scale [13,14]. RPE was recorded at three standardized time points: Pre-intervention, following completion of the standardized warm-up and immediately prior to the maximal 100-yard swim; Mid-protocol, immediately following the assigned intervention (supplemental oxygen or placebo) and prior to the subsequent 50-yard swim; and Post-exercise, immediately following completion of the 50-yard maximal swim. RPE values were reported verbally by participants and recorded by the investigator.

### 2.7. Statistical Analysis

A target sample size of n = 20 was determined a priori based on the crossover design and anticipated within-subject variability, recognizing the increased statistical efficiency afforded by within-subject comparisons relative to parallel-group designs. This target sample size also allowed for potential attrition and data-quality exclusions. Sample sizes in this range are consistent with prior randomized crossover studies in exercise physiology and supplemental oxygen research, which commonly enroll approximately 12–20 participants when evaluating within-subject physiological and performance responses [15,16].

Descriptive statistics are presented as mean ± standard deviation (SD), with corresponding 95% confidence intervals (CI) where appropriate unless otherwise noted. Prior to inferential testing, outcome data were screened for completeness and extreme values; details of data screening procedures and outlier handling are provided in Supplementary Methods S1. All primary analyses were conducted on the final sample of eighteen participants (n = 18).

Normality of paired differences was assessed using Shapiro–Wilk tests. The primary performance outcome (50-yard sprint time) was analyzed using paired-samples *t*-tests comparing the supplemental oxygen and placebo conditions. Within-subject effect sizes were calculated using Cohen’s dz and interpreted using conventional benchmarks (0.20 small, 0.50 medium, 0.80 large) [17,18]. To assess robustness to distributional assumptions, a non-parametric Wilcoxon signed-rank test was additionally performed for the primary performance outcome.

Ratings of perceived exertion (RPE) were analyzed using a 2 (condition: control, intervention) × 3 (time: pre, mid, post) repeated-measures analysis of variance (ANOVA). When significant main effects were identified, post hoc paired comparisons were conducted using Holm-adjusted *p*-values to control for multiple testing. Effect sizes for ANOVA outcomes were reported as partial eta squared (partial η^2^), and within-time-point paired comparisons were summarized using Cohen’s dz (0.20 small, 0.50 medium, 0.80 large) [17,18].

All statistical tests were two-tailed, with statistical significance set a priori at α = 0.05. Analyses were performed using R version 4.5.0 (R Foundation for Statistical Computing, Vienna, Austria).

## 3. Results

Eighteen trained male aquatic athletes were included in the final analyses following a priori data screening (Supplementary Methods S1). All participants completed both experimental conditions and no adverse events were reported. Descriptive statistics, inferential outcomes and effect sizes for all performance and perceptual variables are summarized in Table 2.

### 3.1. 50-Yard Maximal Swim Performance

Fifty-yard sprint performance was significantly improved under the intervention condition compared with control. Mean sprint time was 32.91 ± 3.15 s in the control condition and 31.92 ± 2.22 s in the intervention condition. A paired-samples *t*-test revealed a significant reduction in sprint time under the intervention (mean difference = −0.99 ± 1.67 s; 95% Cl: −1.82 to −0.16 s; t(17) = −2.51, *p* = 0.023), corresponding to a moderate effect size (Cohen’s dz = −0.59). A non-parametric Wilcoxon signed-rank test confirmed this finding (*p* = 0.030).

The intervention resulted in an average 2.67% improvement in sprint performance, indicating a meaningful enhancement in short-distance speed.

### 3.2. Ratings of Perceived Exertion

Ratings of perceived exertion were analyzed using a 2 (condition: control, intervention) × 3 (time: pre, mid, post) repeated-measures ANOVA. There were a significant main effect of time (*p* < 0.001) and a significant main effect of condition (*p* = 0.013). The condition × time interaction was not significant (*p* = 0.99). Post hoc paired comparisons indicated reduced RPE in the intervention condition at the post-exercise time point (*p* = 0.017). No differences were observed between conditions at the pre-exercise (*p* = 0.145) or mid-protocol (*p* = 0.074) time points.

## 4. Discussion

Supplemental oxygen during exercise has produced equivocal performance benefits in prior research, while its effects during recovery intervals between repeated maximal efforts remain underexplored, particularly in swimming where ventilatory constraints may amplify recovery limitations [9,10]. The present study tested whether brief hyperoxic recovery (98% O_2_) following a maximal 100-yard swim improves subsequent 50-yard performance and RPE in trained aquatic athletes. Such outcomes hold practical importance for sprint swimming training, where repeated maximal efforts with short recovery are common and even small performance improvements can prove decisive in competitive contexts.

This study examined whether brief inhalation of 98% supplemental oxygen during acute recovery following a maximal 100-yard swim effort influences subsequent 50-yard freestyle swimming performance and perceived exertion in trained aquatic athletes. The primary findings were that oxygen-assisted recovery was associated with faster subsequent 50-yard maximal swim performance (mean paired difference −0.99 s; 95% CI −1.84 to −0.14 s; Cohen’s dz = −0.59, medium effect) and reduced perceived exertion at the post-exercise time point compared with placebo conditions. Collectively, these findings extend prior recovery-targeted oxygen research into an aquatic setting and support the broader concept that the ergonomic value of hyperoxia may be greatest when timed to the recovery interval rather than that demanded during exercise itself [1,2,10].

### 4.1. Performance Effects

A key feature of the present protocol is that oxygen was delivered only during recovery, so the observed performance difference is more consistent with altered recovery kinetics than enhanced oxygen delivery during the swim itself. This aligns with repeated maximal-effort physiology showing that the ability to reproduce maximal power output is strongly constrained by short-interval restoration processes rather than by aerobic capacity alone [4,5].

From a mechanistic standpoint, the most plausible metabolic explanation remains oxygen-sensitive restoration of high-energy phosphates. Phosphocreatine resynthesis is oxygen dependent, with a substantial portion of recovery occurring rapidly early in the post-exercise period; experimental work shows that limiting oxygen availability slows PCr recovery kinetics in trained humans [5,6]. In the present protocol, the combination of a maximal 100-yard effort, a brief recovery interval, and an immediate subsequent maximal 50-yard effort creates conditions where incomplete PCr restoration is a credible limiting factor of the second effort. Thus, augmenting oxygen availability during that recovery window provides a physiologically coherent pathway to faster subsequent performance, especially in a sport where athletes cannot freely ventilate and normalize CO_2_/H^+^ dynamics during and immediately after maximal efforts [2,6,9].

Importantly, the magnitude of improvement was not uniform across athletes (i.e., median improvement smaller than the mean), indicating heterogeneity of responsiveness. Despite an overall medium effect size (dz = −0.59), individual responses varied considerably, likely reflecting differences in event specialization, recovery capacity, and ventilatory constraints. This pattern suggests the group-level benefit was driven by larger improvements in responsive athletes rather than uniform small gains across all participants. Thus, even small-time differences can be meaningful in short-distance swimming outcomes, where competitive placement can hinge on very small margins [19].

### 4.2. Perceptual Effects

In parallel with the observed performance effects, oxygen-assisted recovery was associated with lower perceived exertion after the intervention was received at the post-exercise time point, with no significant differences observed at pre-exercise or mid-protocol timepoint. Importantly, perceived exertion did not differ between conditions prior to the initial 100-yard swim, indicating that oxygen supplementation did not alter baseline readiness or anticipatory perception. Rather, the divergence in RPE emerged only after exposure to the recovery intervention and subsequent exertion, suggesting that oxygen availability during recovery influenced how fatigue was experienced during the subsequent task execution. This pattern is consistent with a generalized attenuation of perceived fatigue following recovery rather than an isolated effect at a single time point, aligning with prior work demonstrating hyperoxic breathing conditions can shift air-hunger responses and with the established role of RPE as an integrative marker of physiological and perceptual fatigue during high-intensity exercise [9,13,20,21].

We posit that a plausible physiological explanation is that hyperoxia during recovery can reduce dyspnea-related sensations (e.g., “air hunger”) and the subjective burden of ventilatory drive, which is particularly relevant to swimming where breathing is mechanically and behaviorally constrained [22]. Because RPE is an integrative perceptual signal that tracks physiological strain across contexts, even modest shifts can be practically meaningful when combined with improved performance, as they imply athletes may tolerate repeated maximal efforts with less subjective cost [10,20].

### 4.3. Implications in Aquatic Sports and Training

The underwater environment and swimming-specific respiratory mechanics may impose cardiopulmonary constraints that differ from many land-based modalities, including hydrostatic pressure effects and breathing pattern restriction, factors that can prolong ventilatory strain and delay full perceptual recovery between maximal efforts [1,2]. Hyperoxia literature in healthy athletes has repeatedly emphasized context dependence and mixed findings when oxygen is applied during exercise; by contrast, timing oxygen to recovery is increasingly recognized as the more mechanistically defensible window for influencing subsequent high-intensity performance [6,10]. The present findings support that framework and suggest that swimming, because it is inherently recovery-limited by ventilation, may be a particularly relevant testbed for recovery-targeted oxygen strategies [2].

Practically, these results point toward a potential tool for repeated maximum-intensity training sets where the limiting factor is the ability to reproduce maximal efforts with short rest. In swimming, this situation commonly arises during sprint-oriented sets, race-pace training, or repeated maximal efforts within a single session, where incomplete recovery can rapidly degrade performance quality and technical execution. Although the present study was conducted in an aquatic setting, similar recovery constraints exist in other training contexts characterized by clustered maximum-intensity bouts, limited recovery windows, and accumulating perceptual strain across a session. The combination of faster subsequent performance and reduced post-effort RPE is consistent with improved readiness for repeated maximal efforts, an outcome relevant to training quality and fatigue management in sprint-oriented swim sessions. This type of applied, field-feasible intervention also fits the broader push for ecologically valid sport science conducted in real training environments [23].

### 4.4. Limitations

Several limitations should be considered. First, manual stopwatch timing introduces measurement error; although the crossover design reduces bias, future work should use automated timing. Second, mechanistic inference is limited by the absence of physiological recovery measures (e.g., muscle oxygenation, ventilation, blood lactate, or direct PCr kinetics). Given the proposed recovery mechanism, incorporating such measures would directly test whether oxygen accelerates recovery indices that plausibly mediate the second effort. Third, although the placebo was designed to mimic the sensory experience of oxygen delivery, expectancy effects cannot be fully excluded, especially for perceptual outcomes. In addition, the peppermint flavoring used in both conditions may itself influence breathing comfort or perceived exertion independent of oxygen delivery, which could contribute to perceptual differences despite identical sensory exposure. Future studies should consider including a non-flavored control condition or assessing participants’ perceptions of the intervention to further isolate physiological from sensory effects. Finally, all participants in the present study were trained young male athletes, which limits the generalizability of these findings to female athletes, older populations, and untrained individuals. Given known sex- and age-based differences in respiratory mechanics, fatigue perception, and recovery physiology, future work should examine whether recovery-targeted oxygen supplementation produces similar effects in older, female, mixed sex athletic, and untrained populations.

## 5. Conclusions

Brief inhalation of 98% supplemental oxygen at the onset of recovery after a maximal 100-yard swim was associated with faster subsequent 50-yard performance and reduced post-exercise perceived exertion in trained aquatic athletes. These findings support recovery-targeted hyperoxia as a timing-dependent strategy and extend the oxygen supplementation literature into swimming, where constrained ventilation may amplify the importance of recovery processes between maximal efforts.

## Figures and Tables

**Table 1:** Participant Demographics and Anthropometric Characteristics (n = 18).

Variable	Mean ± SD	Range
Age (years)	20.5 ± 1.4	18–23
Height (cm)	183.0 ± 9.4	170.2–200.4
Body Mass (kg)	87.1 ± 15.3	70.5–126.7
BMI (kg·m^−2^)	26.0 ± 3.5	21.8–35.8

All participants were male. BMI = body mass index.

**Table 2:** 50-Yard Maximal Swim Performance and Ratings of Perceived Exertions Under Placebo and Supplemental Oxygen Conditions.

Outcome	Placebo	Supplemental Oxygen	Mean Difference (95% CI)	*p*-Value	Effect Size
50-yard swim time (s)	32.91 ± 3.15	31.92 ± 2.22	−0.99 (−1.84, −0.14)	0.023	−0.59
RPE—Pre	9.78 ± 2.21	8.78 ± 1.56	−1.00 (−2.35, 0.35)	0.145	−0.34
RPE—Mid	17.56 ± 1.84	16.94 ± 1.73	−0.62 (−1.31, 0.06)	0.074	−0.45
RPE—Post	18.78 ± 1.48	17.67 ± 1.59	−1.11 (−1.92, −0.30)	0.017	−0.69

Values are mean ± SD. Mean differences represent supplemental oxygen minus control. Effect sizes are reported as Cohen’s dz for paired comparisons, interpreted per conventional benchmarks (0.20 small, 0.50 medium, 0.80 large); negative dz indicates faster swim times with supplemental oxygen intervention. *p*-values are from paired-samples *t*-tests; RPE values reflect Holm-adjusted post hoc comparisons following repeated-measures ANOVA. n = 18. All participants were male.

## Data Availability

The original contributions presented in the study are included in the article/Supplementary Material, further inquiries can be directed to the corresponding author.

## Supplementary Materials

The following supporting information can be downloaded at: https://www.mdpi.com/article/10.3390/sports14030085/s1, Supplementary Methods S1: Data Screening and Outlier Handling.

## Abbreviations

The following abbreviations are used in this manuscript:

PCr	Phosphocreatine
RPE	Rating of Perceived Exertion
UCLA	University of California, Los Angeles
