# Supplementary material for: Recovery-Targeted Supplemental Oxygen Enhances Performance and Attenuates Perceived Fatigue During Subsequent High-Intensity Swimming
Source: Sports (Basel). 2026 Feb 24;14(3):85. doi: 10.3390/sports14030085 (PMC13030180; doi:10.3390/sports14030085)
Supplement: Supplementary file 1 [file sports-14-00085-s001.zip › Supplementary Methods S1.pdf]

Supplementary Methods S1.

Data Screening and Outlier Handling

Prior to inferential analysis, outcome data were screened for completeness and extreme values. Two participants (IDs 19 and 20) exhibited 50-yard sprint times that were identified a priori as extreme outliers based on inspection of paired difference distributions and standardized residuals exceeding ±3 SD from the sample mean. These values were not consistent with the remainder of the cohort and were deemed likely to reflect non-representative performance unrelated to the experimental manipulation. Accordingly, these participants were excluded from all analyses prior to hypothesis testing.

All primary analyses were therefore conducted on a final sample of eighteen participants using a within-subject crossover framework. To assess the robustness of the primary performance outcome, a non-parametric Wilcoxon signed-rank test was additionally performed, which confirmed the direction and statistical significance of the paired difference observed in the parametric analysis. Effect sizes were reported using Cohen's dz to reflect within-subject standardized differences.

This approach was selected to balance sensitivity to within-subject effects with transparency regarding data handling, while minimizing undue influence of extreme observations on mean-based estimates.
